# Lockdown and adolescent mental health: reflections from a child and adolescent psychotherapist

**DOI:** 10.12688/wellcomeopenres.15961.2

**Published:** 2021-02-17

**Authors:** Jocelyn Catty

**Affiliations:** 1 Tavistock & Portman NHS Foundation Trust, London, NW3 5BA, UK

**Keywords:** Adolescent mental health, psychoanalytic psychotherapy, COVID-19 pandemic, deliberate self-harm, quarantine, temporality in health care

## Abstract

The author, a child and adolescent psychoanalytic psychotherapist working in the UK NHS, discusses the varied impacts of ‘lockdown’ on adolescents, their parents and the psychotherapists who work with them, during the COVID-19 pandemic, in this short observational paper that contributes to the *Waiting in Pandemic Times* Wellcome Collection in response to COVID-19. She asks, particularly, how psychological therapies are positioned during such a crisis, and whether the pressures of triage and emergency can leave time and space for sustained emotional and psychological care. She wonders how psychoanalytic time with its containing rhythm can be held onto in the face of the need for triage on the one hand and the flight to online and telephone delivery on the other. Above all, the author questions how the apparent suspension of time during lockdown is belied by the onward pressure of adolescent time, and how this can be understood by, and alongside, troubled adolescents.

## Introduction

The time of the COVID-19 virus brings a strange shifting of priorities to my professional life as a child and adolescent psychoanalytic psychotherapist working in a Child and Adolescent Mental Health Service (CAMHS) in the UK. COVID-19: the name itself encapsulates delay ( Flexer, 2020, *Waiting in Pandemic Times*). Building into the term the origins of the virus in 2019, it provides a stark reminder that, having ignored warnings from the medical world and then the evidence before our eyes, we are now always already trying to catch up ( Horton, 2020).

The world is in crisis, but it is hard to position the acute and chronic crises of mental health work in the National Health Service (NHS) against the unfolding crisis we see on our screens. Are we high priority or low? Frontline or routine? Do we, like primary care staff, rush to ‘man the barricades’ ( Davies, 2020, *Waiting in Pandemic Times*) – anxiety about the possibility of redeployment is spreading among mental health staff even where they are entirely untrained for physical health care – or do we hunker down at home to conduct therapy online for the foreseeable future? And what is foreseeable about the future, now, for the young patients, depressed, anxious or enduring the turbulence of adolescence, for whom the future was only hazily in view in the first place?

Mental health has traditionally been lamented as the poor relation within the NHS, with psychiatry under-valued and repeated cries to achieve parity between mental and physical health ignored. How, then, are we to consider the seriousness of psychological and emotional labour conducted in services such as CAMHS during a national crisis? Talking to young people and children about their anxieties, or even their considerable distress, appears low priority when compared to doctors and nurses battling COVID; yet an adolescent death by suicide remains one of the most catastrophic events imaginable, for family, friends and professionals alike. In the time of the virus, we are thus adrift in the prevailing geo-spatial metaphors of the age: nowhere near the ‘front line’, we may find ourselves thrust suddenly towards it if a teenager makes a serious or dangerous attempt to self-harm or commit suicide.

## Adolescent time

The world gives the impression of having halted what we might call ‘adolescent time'. Exams in the UK are cancelled; school is out, or virtual; universities have sent their students home. For those in their teens, the COVID-19 pandemic arrives at a crucial time in development, as they transition from childhood to adulthood. Yet the time of adolescence itself often feels both chronic and acute, its difficulties regarded as perennial, even predictable, yet often plunging the young into crisis. Disturbed adolescents may try to arrest a march of time that feels relentless by retreating into depression, or into their bedrooms: to halt their progress towards a future that is perceived as bleak, or simply unimaginable.

What can we learn about time – now, in the time of COVID-19 – from this sudden suspension of time which is not actually a suspension at all? This questioning of the future which is, curiously, so familiar to many of the young people whose mental health elicits our care?

The decision to award GCSE and A level results in the summer of 2020, rather than postpone the exams, could be seen as a shocking pronouncement: that time waits for no one, that adolescent progress cannot, must not, be halted – even if, for those awarded a grade less than that which they might have achieved, progress is thwarted. Like their younger counterparts at the top of primary school, they must, even from their bedrooms, be ushered forwards to the brink at which they bid their school lives farewell. Those struggling with the pressure of work and exams may be relieved, but their world has also crashed down upon them and many are disappointed. Some lament a lack of control: the final academic effort, for which they were preparing, is denied them, and teachers, or government, will decide upon their grades. Yet for some, for whom the pressure of external life has been unbearable, perhaps there is the possibility of respite, and the lockdown may provide them with much-needed time for recovery.

Adolescent development ‘runs unevenly’ ( Waddell, 2018, p. 26): how the time of COVID-19 intersects into each individual trajectory will vary hugely. While some sections of the media portray the young as oblivious – gathering in parks, spitting defiantly in the faces of police or the elderly – we hear our young patients report their varying responses, almost always ambivalent, anxious. For those with depression, existential despair, sometimes born of inter-generational trauma and loss, is known to dominate ( Catty ed., 2016): how are they to believe that the future holds any promise when it appears to have been cancelled, or at least indefinitely postponed? For some, this will confirm a pre-existing belief, a bleakness. Meanwhile, they worry about grandparents, parents and, increasingly, each other.

There is an idea that psychoanalytic work with adults involves the recollection and processing of remembered trauma – that it is, as Wordsworth wrote of poetry, ‘emotion recollected in tranquillity’ ( 1805/1987, p. 42) – while therapy with children and adolescents is conducted during and alongside the unfolding of their key emotional dramas. Theory and clinical practice afford many contradictions of this dichotomy; yet it remains meaningful to conceptualise adolescent therapy as a ‘being alongside’ a teenager as they live through what can feel like their most turbulent of times. How does lockdown impact on this sense of immediacy? During lockdown, young people are suffering a crisis that we appear to share with them, at least in this basic way: we too sit in our homes as we engage them in their therapy. Keeping a focus on the particularity of their experience – the extent to which the national crisis may or may not be impacting on their internal dramas – will need close attention. Yet perhaps they have something to tell us about uncertainty – about the future, about the passing of time – that they have long feared we did not understand. For some, we have finally entered into their world. There are implications here, too, for our work with their parents, now that we feel ourselves to share their most immediate circumstances: we are all in lockdown; we are all worried about our ageing parents; we are all, increasingly, worried about the young.

## Urgency and delay

Crisis time in adolescent mental health services relies on a red-amber-green system of case-flagging. Now only the reddest of the red cases can be seen in person, anxiously diverted from Accident and Emergency departments to the community clinic to avoid contamination. While those on duty manage these most critical of crises in person, the rest of the team connect to their patients via telephone and video-conferencing. Fears that mental health work will be deemed such low priority as to justify sending therapists into the medical settings for which they would be entirely, shockingly, unprepared, seem to abate as authorities determine that mental health emergencies are themselves ‘priority’. At the same time, the urgency of attending to an unfolding mental health crisis is becoming clearer: articulated in a recent ‘call for action’ to include data collection on the psychological, social and neuroscientific effects of the pandemic on both the general population, vulnerable groups and those with the virus ( Holmes *et al.*, 2020).

What, then, are the implications of mental health triage in this new world? In the early weeks of the lockdown, we wonder whether to activate a crisis response by focusing only on emergencies, keeping in touch with our regular patients for more frequent, but briefer, telephone updates. Implicitly, we are invoking ideas of triage (focusing only on emergencies in any detail or depth) and support (finding out how our patients are managing, rather than working with them). Yet it is clear that such a model will not serve us well in the longer-term: if nearly the whole CAMHS population is provided with brief, intermittent support rather than treatment, logic dictates that their mental health will deteriorate. Yet does such a distinction between support and treatment hold in a time of crisis? It is a distinction that has always been uncomfortable where it privileges the activity of psychological therapists over other mental health specialisms, such as nursing, occupational therapy or social work (deemed to be providing ‘support’ or ‘risk management’); yet it has enabled us to retain an emphasis on the ‘work’ that is involved in psychological treatment and the process that unfolds between the participants in psychotherapy, patient and therapist. What the nature of such work may be during lockdown remains to be seen.

Meanwhile, mental health emergencies among the teenage population seem to have plummeted: we wonder, where are they? Have they too been suspended? There is anxiety about when the dam may break; an increase in anxiety, depression and self-harm is expected in the population as a whole ( Holmes *et al.*, 2020) ^1^. For those that come in, we find ourselves contorting the familiar NHS language of ‘risk’: do we mean suicide risk or COVID-19 risk? Where is ‘safe’ for a 16 year-old determined to kill herself, or a 13 year-old who has taken an overdose? A mother asks whether, were her teenage son to harm himself, she would be allowed to be with him in hospital; we cannot advise her. The focused maternal care that a teenager may specifically crave in such desperate moments becomes the one thing he would deprive himself of; the choices facing those with suicidal thoughts become starker now. We ask ourselves, can we provide a reassuring presence dressed in protective mask and goggles? Or should we retreat behind a computer or smartphone screen, through which we can, at least, be seen as ourselves?

## Time and space

How do we keep time in such a crisis? There is a rhythm that psychotherapists and their patients come to live and breathe: the regular pulse of the psychoanalytic session, whether weekly or more frequent; the predictability of the starting time; the inevitability of the session end or the week’s wait. This rhythm underpins the duration of a therapy as it unfolds in time and is the bedrock of the ‘containment’ ( Bion, 1962) that psychoanalysis offers ( Baraitser & Salisbury, 2020, *Waiting in Pandemic Times*). Can this rhythm, based on the fifty-minute hour, be maintained over the telephone or protected with the same boundaries as in the clinic?

In the rush of psychotherapists to online platforms and the telephone, can we maintain this steady pulse? For a teenage patient, does it still feel like his session time if he knows his therapist is going to ring? Will it still feel like time to stop if we are wrapped in the cocoon of sound provided by a telephone call in a quiet room; or if we have been trying to focus on each other’s faces in a shaky video call? Despite the fact that most teenagers are more familiar with online discourse than we are, this shift raises issues of space too. Is it intrusive to conduct therapy online with an adolescent, looking into that most private of spaces, their bedroom? Alternatives are unlikely when families are crammed together conducting school and home lives under one roof. What is it like for a depressed adolescent to know that his therapist is telephoning from her own home? Or for a troubled teenage girl, reliant on self-harm to embody her misery, to bring her therapist into her home on a smartphone screen?

Decisions continue to need making: despite the impression that time has been suspended, in fact it waits for nobody. An offer of time-limited psychotherapy for a girl of seventeen-and-a-half is paused: can it still be done? The time-frame provided by the therapy model was to fit neatly into the time that remains for her as a CAMHS patient: upon her eighteenth birthday, she will be discharged. Despite the impression that the world has stopped turning, time is marching on.

Nothing sums up better the paradox of the crisis for adolescents or gives the lie more obviously to the notion of shutdown, suspension or postponement. Time is still passing.

### After-word

This observational paper was written during the first few months of lockdown, between March and May 2020, as part of a collective attempt by the author and scholars of the Waiting Times project to reflect on the implications of the pandemic and the UK government’s response to it in the form of a complete national lockdown. Returning to it nine months later, with the twin benefits of peer review and the passage of time, a number of reflections come to mind.

First, it is striking how little choice I felt myself to have over the kind of writing I could engage in during that crisis time: the keener analysis that might come with time was, to me then, impossible. This was not simply for ‘time reasons’. It was perhaps not very different from the frequent complaint from others in lockdown, particularly those whom furlough, redundancy, or the lack of a commute had given them time, that they felt unable to settle down to read, or to write. Yet in the face of the onslaught of anxiety occasioned by the arrival of COVID-19, it felt crucial to be able, somehow, to think. Given all the war analogies offered during the first wave of the pandemic in the UK, it is perhaps fitting to think back to Virginia Woolf, who wrote during World War II that the sound of air raids ‘is a sound that interrupts cool and consecutive thinking about peace. Yet it is a sound…that should compel one to think about peace’ ( Woolf, 1940). In the face of a crisis, perhaps I fell back on one of the core principles of my clinical training: observation ( Sternberg, 2005). The paper was an attempt to share my observations of those early weeks in lockdown from my perspective as a child psychotherapist working in CAMHS, and thus to offer some space for thought.

Second, it is striking how universalising those early weeks of lockdown seemed, as though I had bought into the UK government’s widespread claims that we were ‘all in the same boat’. The impression I wrote about, of sharing in the experiences of my young patients and, particularly, their parents, was rather unmediated. While it is true that the shared experience of the pandemic continues to have a resonance for therapeutic work, it is also true that the pandemic is having tragically differential impacts on different socio-economic, professional and ethnic groups.

Third, as reviewers of this paper observed, there was a tone of pessimism which may or may not have been warranted: pessimism about the potential efficacy of moving psychotherapy for young people online, and about a preponderance of triage rather than treatment, and anxiety about the possibility of a surge in mental health emergency referrals as the effects of both the virus and the lockdown took effect. Looking back in the new year of 2021, with cases and deaths rising, it is hard not to retain some of that pessimism of May 2020. Yet there is also some room for optimism.

The amount of mental health work it has been possible to do in CAMHS services, even while so under-resourced, has been extraordinary; my fears that perhaps only triage would be available have not been realised, although inevitably this has varied nationally and depending on the patient’s age and presentation. Some teenagers have found they prefer the intimacy of the telephone, or the convenience of the video-call, in a way that facilitates rather than hampers clinical work. And the psychological professions have clearly more than risen to the challenge of online work, finding ways to manage the boundaries of time and space in new ways. (While some sessions are compromised by a young person answering the phone in the supermarket, more are sustained in a private space recreated at home.) Whether this is sufficient to off-set all the impacts of lockdown on young people is another matter, of course. It is now widely argued that the pandemic is having devastating impacts on children and young people [ https://www.theguardian.com/commentisfree/2021/jan/16/letters-our-children-are-in-crisis-and-need-help accessed 29.01.21].

As for any consequent tsunami of referrals, pent up after the diminished activity of the first lockdown, or in response to bereavement, trauma or catastrophic anxiety, it is perhaps still too early to tell. It has already become controversial, however. Concern remains very high, evidenced in rapid increases in referrals once schools re-opened in the autumn, and in children’s commissioner Anne Longfield’s warning that CAMHS services are unable to meet children’s mental health needs, with one in six children likely to have a diagnosable mental health condition even by July 2020 [ https://www.childrenscommissioner.gov.uk/2021/01/28/damage-to-childrens-mental-health-caused-by-covid-crisis-could-last-for-years-without-a-large-scale-increase-for-childrens-mental-health-services/ accessed 29.01.21]. The idea that a tsunami of referrals is coming, however, has been challenged by Richard Bentall and the COVID-19 Psychological Research Consortium, who found evidence of resilience and adaptation to lockdown, with adverse outcomes being differentially experienced by those with existing mental health problems and health anxiety; indeed, they argue that ‘the “tsunami” narrative carries the risk of becoming a self-fulfilling prophecy’ [ https://www.theguardian.com/commentisfree/2021/feb/09/pandemic-mental-health-problems-research-coronavirus accessed 09.02.21]. Their rebuttal responds to, inter alia, the claim by the Royal College of Psychiatrists that the pandemic represents ‘the greatest threat to mental health since the second world war’ [ https://www.theguardian.com/society/2020/dec/27/covid-poses-greatest-threat-to-mental-health-since-second-world-war accessed 29.01.21]. Indeed, even the Second World War analogy is a problematic one. Fears that the Blitz in the UK would lead to mass panic - three to four million cases of acute panic and hysteria were predicted - proved unfounded ( Burns, 2013, pp 130–131); hence, indeed, the concept of ‘Blitz spirit’ that has been so mis-used during the current pandemic.

There is much to learn here. Perhaps the most striking thing about returning to this paper in February 2021 is the enormity of the realisation that the UK is in lockdown again and in the grip of a second wave deadlier than the first. Time is still passing, but the impact of a lost year from the lives of young people may be felt for a long time.

## Data availability

All data underlying the results are available as part of the article and no additional source data are required.

## Author information

Dr Jocelyn Catty is Principal Child and Adolescent Psychotherapist in Bromley CAMHS and Research Lead for the Child Psychotherapy Doctoral Training at the Tavistock and Portman NHS Foundation Trust. She is also Senior Research Fellow on the Waiting Times project. She has a DPhil in English Literature; is an adult psychodynamic psychotherapist; and was formerly Senior Research Fellow in Mental Health at St George’s, University of London.
