# Supplementary material for: Lockdown and adolescent mental health: reflections from a child and adolescent psychotherapist
Source: Wellcome Open Res. 2021 Feb 17;5:132. Originally published 2020 Jun 10. [Version 2] doi: 10.12688/wellcomeopenres.15961.2 (PMC7424913; doi:10.12688/wellcomeopenres.15961.2)
Supplement: Peer review history

# Peer review: Reviewer response for version 2

**DOI:** 10.21956/wellcomeopenres.18365.r42676

**Published:** 2021-02-25

**Authors:** Randi Ulberg, Thea Schønning

**Affiliations:** 1 Division of Mental Health and Addiction, University of Oslo, Oslo, Norway; 2 Department of Psychiatry, Diakonhjemmet Hospital, Oslo, Norway; 3 Faculty of Medicine, University of Oslo, Oslo, Norway

No further comments.

Is the work clearly and accurately presented and does it cite the current literature?

Partly

If applicable, is the statistical analysis and its interpretation appropriate?

Not applicable

Are all the source data underlying the results available to ensure full reproducibility?

No source data required

Is the study design appropriate and is the work technically sound?

Partly

Are the conclusions drawn adequately supported by the results?

Partly

Are sufficient details of methods and analysis provided to allow replication by others?

Partly

Reviewer Expertise:

Professor MD, Randi Ulberg: Youth psychotherapy researcher. Internet-based youth psychotherapy. Medical student Thea Schønning: Young advisor (PPI)

We confirm that we have read this submission and believe that we have an appropriate level of expertise to confirm that it is of an acceptable scientific standard.

# Peer review: Reviewer response for version 1

**DOI:** 10.21956/wellcomeopenres.17505.r39213

**Published:** 2020-08-12

**Authors:** Randi Ulberg, Thea Røstbakken

**Affiliations:** 1 Faculty of Medicine, University of Oslo, Oslo, Norway; 2 Division of Mental Health and Addiction, University of Oslo, Oslo, Norway; 3 Department of Psychiatry, Diakonhjemmet Hospital, Oslo, Norway

The child and adolescent psychoanalytic psychotherapist, Jocelyn Catty reflects on how psychological therapies are positioned during a crisis such as the COVID-19 pandemic. The author questions how the psychoanalytic session is maintained over online platforms and telephone consultations. Furthermore, Catty addresses essential reflections of how the crisis leaves time for emotional and psychological care in a time characterized by the pressure of triage and emergency. The introduction outlines the immediate and dark consequences for young people, their school achievements, mental health, social development, and the opportunity to get adequate treatment. She points at the specific developmental challenges of COVID-19 putting young people at high risk for lagging behind in this important transitional phase into adulthood. 

 The manuscript is well-written and contains highly needed questions regarding adolescent mental health during a crisis as the pandemic. It emphasizes the uncertainness in therapy rooms and improvised therapy rooms at home worldwide. The paper raises the urgency for young people and the need for society to take their situation seriously. Jocelyn Catty fears that only mental health triage will be offered to young people and a generation will be deprived of the opportunity for treatment. The paper is formulated as a warning bringing into the discussion some problematic sides of video consultations.

 The paper is submitted as a research article, however, we read it to be an opinion article. Thus, evaluation according to research standards is not applicable. The paper does not provide sufficient details of method and analysis to allow replication of others, neither are any conclusions drawn - as there are no results and the manuscript is lacking both qualitative and quantitative data. However, as an opinion article, the paper is a structured and well written, article.

 However, the manuscript might be more useful to a broad clinical readership if the author moved beyond the rather pessimistic undertone regarding psychotherapy during a crisis and explored alternative perspectives. As there is evidence as well as clinical experiences during the ongoing pandemic that some young people in some periods of therapy might profit even more from video consultations. In addition, video consultations give the therapist an opportunity to follow the young person and offer treatment even when students according to change in school or study situation move to other places.

 Some questions the author may consider: How does the therapists’ attitude towards online therapy or telephone consulting affect the therapy delivered on these platforms? How is the therapist marked by the current crisis and shaped by being forced to deliver therapy on alternative platforms? Might it be that the physical distance to the therapist for some adolescents facilitates a greater emotional closeness to the psychotherapy – and therapist? 

 The manuscript ends rather abruptly with loads of important questions to reflect on without stating a clear take-home message. 

 Overall, this is a timely and much-needed essay. It is written nicely with rich metaphor and moving examples of how these last months has changed the entire field of psychotherapy – both for patients and most therapists. The paper might be improved by modifications addressing the detailed comments below and perhaps a mention of alternative perspectives to better recognizing the complexity of this important matter.

Is the work clearly and accurately presented and does it cite the current literature?

Partly

If applicable, is the statistical analysis and its interpretation appropriate?

Not applicable

Are all the source data underlying the results available to ensure full reproducibility?

No source data required

Is the study design appropriate and is the work technically sound?

Partly

Are the conclusions drawn adequately supported by the results?

Partly

Are sufficient details of methods and analysis provided to allow replication by others?

Partly

Reviewer Expertise:

Professor MD, Randi Ulberg: Youth psychotherapy researcher. Internet-based youth psychotherapy. Medical student Thea Røstbakken: Young advisor (PPI)

We confirm that we have read this submission and believe that we have an appropriate level of expertise to confirm that it is of an acceptable scientific standard.

# Peer review: Reviewer response for version 1

**DOI:** 10.21956/wellcomeopenres.17505.r39029

**Published:** 2020-06-15

**Authors:** Tom Burns

**Affiliations:** 1 Department of Psychiatry, Warneford Hospital, University of Oxford, Oxford, UK

This is a provocative and timely article. Catty has used the remarkable interruption of normal CAMHS psychotherapy practice  by the COVID-19 crisis to explore and illuminate the importance of time and 'the future' for adolescents and the, often overlooked, impact of rhythm in the psychotherapeutic process.

 The importance of the article would seem to lie in its engagement with a rapidly changing (and novel) challenge to practice. The trade offs between (masked) face to face sessions with digital but unmasked consultations are well noted.

 Like many writing about COVID Catty appears to accept the 'looming mental health' epidemic it will cause while observing that initially referrals fell. It might be worthwhile to revisit previous national crises (eg. WWII) when predictions of mass psychological casualties were found to be baseless.

 Catty acknowledges that her thinking is based on the first few weeks of lockdown and a folllow up article after a couple of months to compare her thoughts with what transpires would be of considerable interest.

Is the work clearly and accurately presented and does it cite the current literature?

Yes

If applicable, is the statistical analysis and its interpretation appropriate?

Not applicable

Are all the source data underlying the results available to ensure full reproducibility?

No source data required

Is the study design appropriate and is the work technically sound?

Yes

Are the conclusions drawn adequately supported by the results?

Yes

Are sufficient details of methods and analysis provided to allow replication by others?

Yes

Reviewer Expertise:

social psychiatry and the application of psychotherapeutic principles in adult disorders.

I confirm that I have read this submission and believe that I have an appropriate level of expertise to confirm that it is of an acceptable scientific standard.
